# Cannabidiol Treatment in a Predator-Based Animal Model of PTSD: Assessing Oxidative Stress and Memory Performance

**DOI:** 10.3390/ijms26104491

**Published:** 2025-05-08

**Authors:** George Jîtcă, Robert Stoicescu, Erzsébet Májai

**Affiliations:** 1 Department of Pharmacology and Clinical Pharmacy, Faculty of Pharmacy, George Emil Palade University of Medicine, Pharmacy, Science and Technology of Târgu Mureș, 540139 Târgu Mureș, Romania; stoicescurobert42@gmail.com; 2 Department of Toxicology and Biopharmacy, Faculty of Pharmacy, George Emil Palade University of Medicine, Pharmacy, Science and Technology of Târgu Mureș, 540139 Târgu Mureș, Romania; erzsebet.fogarasi@umfst.ro

**Keywords:** cannabidiol, oxidative stress, memory, stress, malondialdehyde

## Abstract

Numerous preclinical and clinical studies indicate that CBD possesses various therapeutic properties, including antipsychotic, analgesic, anticonvulsant, antineoplastic, and antioxidant effects. Recent research has also highlighted its potential anxiolytic effects. This study aimed to evaluate the impact of CBD treatment in a PTSD induction model. To determine CBD’s efficacy, behavioral tests assessing anxiety and memory were conducted. Additionally, two oxidative stress markers were measured to explore its antioxidant properties. Forty adult male rats were used for PTSD induction. The procedure involved exposure to predator odor on day 10, followed by a second exposure on day 20. A secondary stressor, consisting of daily cage partner changes, was also applied. The animals were randomized into four groups: two non-stressed and two stressed groups. CBD was administered at 10 mg/kg. Behavioral effects were evaluated using the open field (OF), elevated plus maze (EPM), novel object recognition (NOR), and Morris Water Maze (MWM) tests. Malondialdehyde and the GSH/GSSG ratio were assessed using liquid chromatography. CBD treatment did not significantly alter anxiety-like behavior in the EPM, though a trend toward increased vertical exploration was observed in the OF test. In memory-related assessments, no significant differences were found in the NOR test, while performance in the MWM indicated improved spatial memory, with CBD-treated rats spending more time in the target quadrant. In addition, malondialdehyde levels decreased in the CBD groups. Elevated cortisol levels in the stressed CBD group suggest a potential anxiolytic effect, warranting further research.

## 1. Introduction

Post-traumatic stress disorder (PTSD) is a condition often developed by people exposed to catastrophic events. Most often, the symptoms developed by these patients include avoidance, mood change, and cognitive alteration. Also, other psychiatric co-morbidities that accompany PTSD include depression, anxiety and substance use disorder. Despite substantial advances in the understanding of PTSD over recent decades, the neurobiological mechanisms underlying the disorder remain poorly understood [1].

In the present study, we resorted to a predator-related stimuli model (scent of predator urine) combined with unstable housing conditions (daily swapping cage partners), leading to a predator-based psychosocial stress paradigm for PTSD induction. This exposure leads to intense fear and stress, followed by behavioral and endocrine changes [2,3]. Moreover, clinical evidence indicates the involvement of oxidative stress in PTSD, as reflected by increased levels of lipid peroxidation products and the reduced activity of antioxidant enzymes [4,5,6,7], leading to high levels of free radicals in the brain; thus, neuronal death may occur [8,9]. An additional layer of complexity in PTSD arises from its impact on cognitive functions, which are not only directly affected by the disorder but may also be further impaired due to oxidative stress resulting from increased free radical production [10].

It is known that PTSD is associated with the dysregulation of the hypothalamic–pituitary–adrenal axis, and patients diagnosed with PTSD show low levels of plasma cortisol [11]. In order to create a general picture of cortisol variance, an integrative model suggests that increased plasma levels are present as an acute response to stress. Furthermore, both low pre-traumatic and reduced post-traumatic cortisol levels have been proposed as potential prognostic biomarkers for PTSD [12]. In reality it is very difficult to capture the exact moments of pre- and post-traumatic events. However, some results suggest that low levels of cortisol, assayed shortly after the traumatic event, might be correlated with PTSD development [13]. Because of the discrepancies in results [14,15,16], it is not known if cortisol can be used as a marker for PTSD.

Pharmacological treatment typically involves selective serotonin reuptake inhibitors (SSRIs), such as paroxetine or sertraline, and is often administered in conjunction with other therapeutic approaches. Evidence shows that combined therapy may be more effective than one medication alone. Also, other compounds, such as cannabidiol (CBD), have gained popularity as a potential anxiolytic treatment [17,18,19]. Many preclinical and clinical studies have suggested that CBD has a broad range of therapeutic properties, such as antipsychotic, analgesic, anticonvulsant, antineoplastic, antioxidant properties [20]. Recent studies have shown the anxiolytic effect of CBD and also suggest a decrease in the salivary cortisol level [21,22]. It is said that these anxiolytic properties are mediated by the interaction with different receptors, including CB1, 5-HT_1A_, TRPV1 [23], but there are also other receptors that CBD is able to modulate, namely PPARγ, GPR55, 5-HT, GABAA. Regarding the antioxidant effects of CBD, these are due partly to its chemical structure, but also to the interaction with molecular mechanisms involved in the regulation of oxidative stress (NRF/KEAP1) [24,25,26]. Studies show that CBD’s effect on cortisol regulation is due to the interaction between the endocannabinoid system and the stress response [27]. The cannabinoid content must also be taken into account, as some can have contradictory effects, increasing cortisol levels, and another factor is the type of use, acute or chronic. Thus, acute use can increase stress hormone levels, while chronic use decreases these hormones [28].

The purpose of our study was to assess the effect of CBD treatment in a PTSD induction model. In order to test the efficacy of CBD for this model, behavioral assessments for anxiety and memory were performed. Also, because we wanted to test the antioxidant properties of CBD, we assayed two markers of oxidative stress.

## 2. Results

### 2.1. Behavioral Tests

#### 2.1.1. Open Field Test (OF)

**Figure 1:**
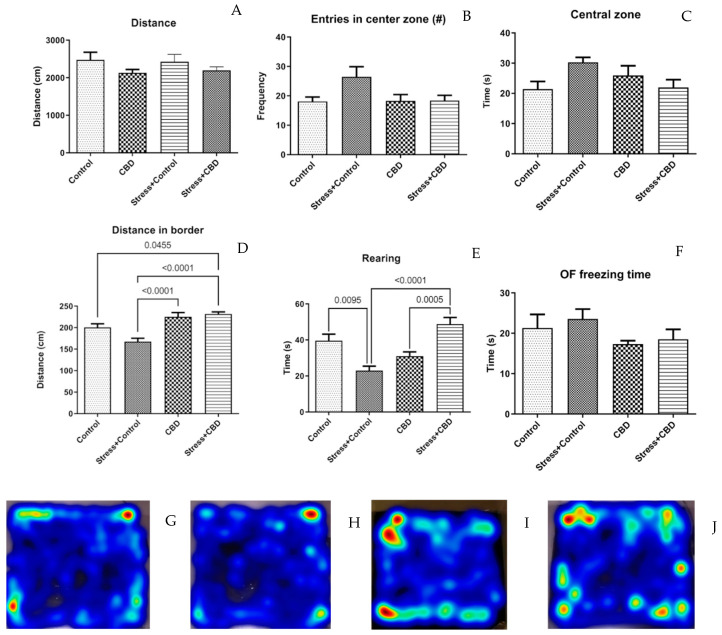
Anxiety-like behavior assessment in the OF test. (**A**) Distance traveled during the trial, F (3, 36) = 1.479, *p* = 0.236. (**B**) Number of entries in the center zone (30 *×* 30 cm^2^), F (3, 36) = 2.910, *p* = 0.0481. (**C**) Time spent in central zone during the trial, F (3, 36) = 1.800, *p* = 0.1652. (**D**) Distance traveled outside the central zone, F (3, 36) = 12.48, *p* < 0.0001. (**E**) Rearing—defined as the vertical exploration measured by counting the number of supported rearing, F (3, 36) = 12.67, *p* < 0.0001. (**F**) OF freezing time—time spent in a freezing state, F (3, 34) = 1.317, *p* = 0.2849. (**G**–**J**) Heatmap of path of all groups. Data are expressed as mean ± SEM.

The OF test revealed no statistically significant differences between the included groups regarding the distance, entries in the center zone, and time spent in the central zone. However, we noticed that some groups spent more time on the border, as can be seen in Figure 1D. Rearing activity relates to the distance on the border, as significant differences can be seen in Figure 1E.

#### 2.1.2. Novel Object Recognition (NOR)

**Figure 2:**
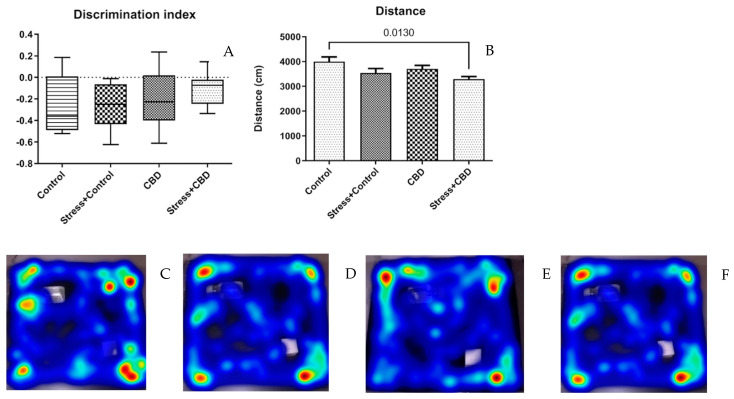
(**A**) Discrimination index and the Kruskal–Wallis test was employed, *p* = 0.2453. (**B**) Distance traveled during the NOR test, (F (3, 36) = 3.871, *p* = 0.0172), (**C**–**F**) Heatmap of path of all groups. Data are expressed as mean ± SEM.

CBD administration did not significantly influence the discrimination index (Stress + CBD—−0.1047 ± 0.04373 vs. CBD—−0.1823 ± 0.07594 vs. Stress + Control—−0.2638 ± 0.07536 vs. Control—−0.2730 ± 0.09653, *p* = 0.2453), as can be seen in Figure 2A. As for the distance traveled in NOR, the Control group displayed a longer distance traveled compared to the Stress + CBD group.

#### 2.1.3. Elevated Plus Maze Test (EPM)

**Figure 3:**
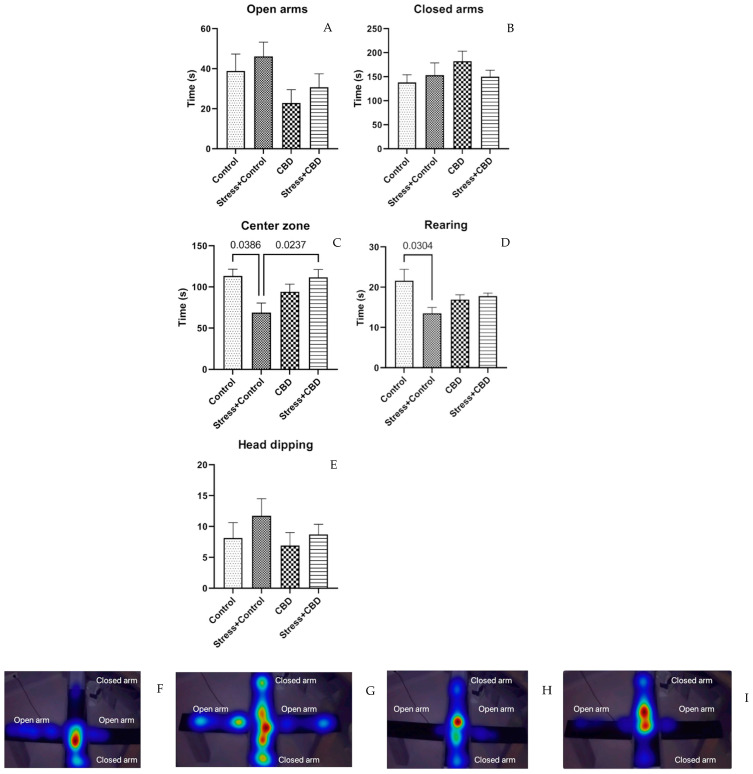
Results from Elevated Plus Maze Test (EPM). (**A**) Time spent in the open arms. (**B**) Time spent in the closed arms. (**C**) Time spent in the center zone. (**D**) Rearing—defined as the vertical exploration measured by counting the number of supported rearing. (**E**) Head dipping—exploratory behavior where a rodent (typically a mouse or rat) lowers its head over the edge of one of the open arms. (**F**–**I**) Heatmap of path of all groups. Data are expressed as mean ± SEM.

The EPM did not reveal any statistically significant difference between the groups for the time spent in opened (*p* = 0.0985) and closed arms (F (3, 36) = 1.097, *p* = 0.3643), Figure 3A,B. As for the time spent in the central zone (F (3, 36) = 3.819, *p* = 0.0188), the Stress + Control group spent a shorter time here compared to the Stress + CBD (Stress + CBD—111.6 ± 9.717 vs. Stress + Control—68.75 ± 11.87, *p* = 0.0237) and Control groups (Control—113.3 ± 8.434 vs. Stress + Control—68.75 ± 11.87, *p* = 0.0386), Figure 3C. The rearing duration also differed between the Stress + Control group and Control group (Control—21.63 ± 2.834 vs. Stress + Control—13.50 ± 1.488, *p* = 0.0304), as can be seen in Figure 3D.

#### 2.1.4. Morris Water Maze (MWM)

**Figure 4:**
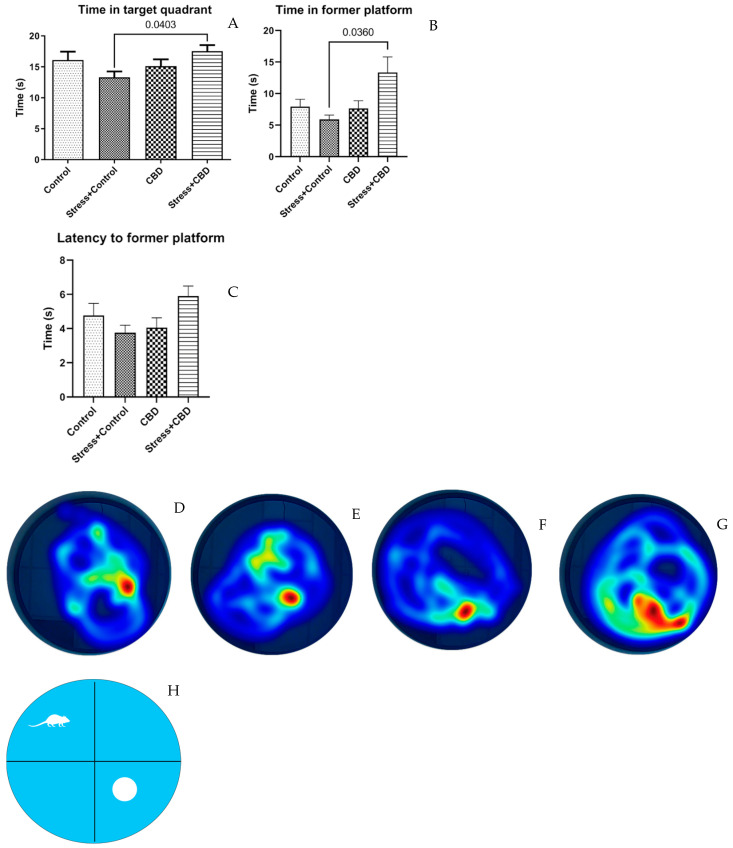
Results from Morris Water Maze (MWM). (**A**) Time spent in the target quadrant. (**B**) Time spent in the former platform. (**C**) Latency to former platform. (**D**–**G**) Heatmap of path of all groups, (**H**) Representation of the MWM. Data are expressed as mean ± SEM.

The time spent in the target quadrant (F (3, 36) = 2.384, *p* = 0.0881) by the Stress + CBD group is significantly longer than that of the Stress + Control group (Stress + CBD—17.52 ± 0.9843 vs. Stress + Control—13.31 ± 0.9496, *p* = 0.0403), Figure 4A. This correlates with the time spent in the former platform place (F (3, 36) = 3.549, *p* = 0.0241), (Stress + CBD—13.37 ± 2.441 vs. Stress + Control—5.906 ± 0.6776, *p* = 0.0360), Figure 4B. It is noteworthy that the latency to the former platform does not differ, with longer time being spent by the Stress + CBD group, F (3, 36) = 2.623, *p* = 0.0664, as can be seen in Figure 4C.

### 2.2. Plasma and Brain Sampling

#### 2.2.1. Plasma and Brain MDA, Plasma GSH/GSSG Ratio

**Figure 5:**
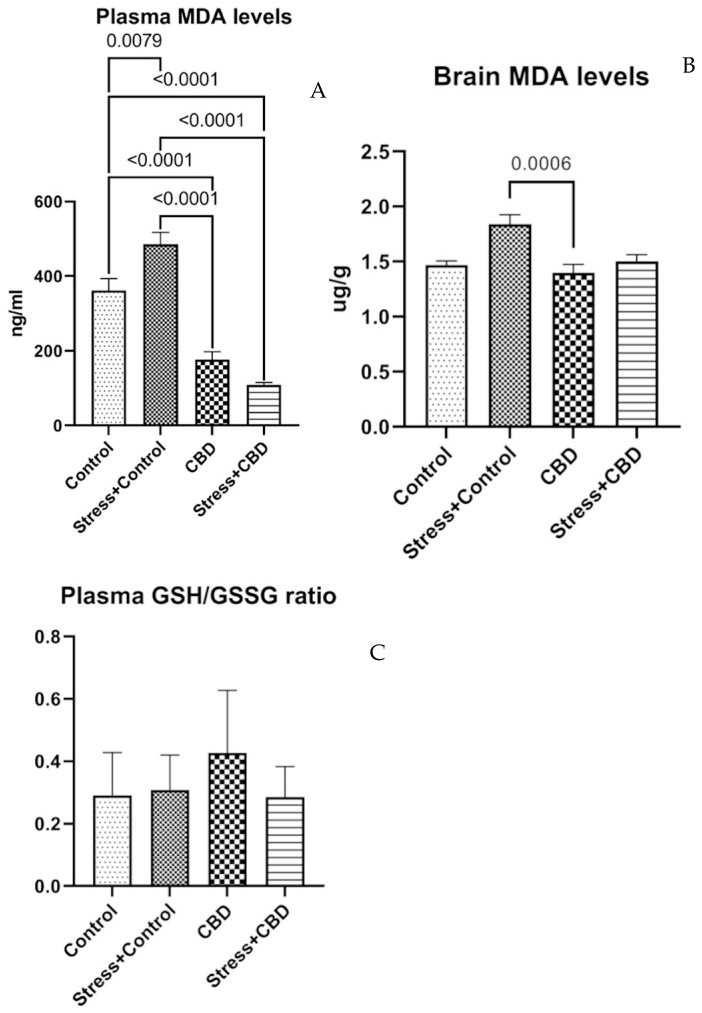
Plasma level of malondialdehyde (MDA) (**A**). Brain level of MDA (**B**). Plasma level of GSH/GSSG ratio (**C**). Data are expressed as mean ± SEM.

The plasma MDA level reveals the differences between the groups included in the study (F (3, 36) = 52.96, *p* < 0.0001). The highest level of MDA is in the Stress + Control group, while the lowest level of MDA in plasma is in the Stress + CBD group. For the brain MDA level, the tendency remains (F (3, 36) = 7.522, *p* < 0.0006), with the Stress + Control group having the highest values as can be seen in Figure 5A,B. The plasma GSH/GSSG ratio reveals no statistical differences, with the CBD group having the highest ratio (F (3, 35) = 2.286, *p* = 0.0958) as can be seen in Figure 5C.

#### 2.2.2. Cortisol Level

**Figure 6:**
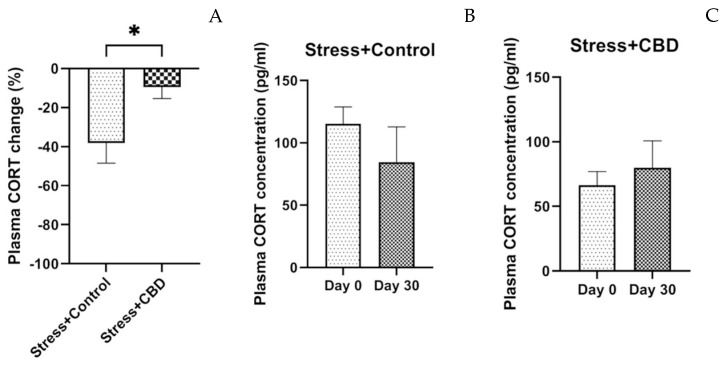
Plasma cortisol (CORT) differences expressed as percentage (**A**), with unpaired *t* test employed. Plasma CORT concentration in Stress + Control group (**B**). Plasma CORT concentration in Stress + CBD (**C**). Data are expressed as mean ± SEM. Paired *t* test was applied for Stress + CBD group and Wilcoxon test was applied for Stress + CBD group. * *p* < 0.05.

The plasma cortisol levels revealed a significant difference between the two groups exposed to stresses. However, repeated exposure to stresses decreased the cortisol level after 30 days in the Stress + Control group, and the cortisol level increased in the Stress + CBD group as can be seen in Figure 6.

## 3. Discussion

Rats are a commonly used species in PTSD research because of the significant similarities in the structure and function of their central nervous system to humans. Both rats and humans respond to stress through activation of the hypothalamic–pituitary–adrenal axis and changes in neurotransmitter activity, making the rat a suitable model for studying the biological mechanisms of PTSD. Rats also allow precise experimental manipulations and can be monitored long term, which is important for studying the chronic effects of post-traumatic stress. The predator-based stress model is relevant to the study of PTSD because it replicates a type of acute and extreme stress similar to the psychological trauma that humans may experience (e.g., through predator attacks or other acute trauma events). This model is effective in inducing a biological response similar to that seen in humans who have experienced major trauma. By using this model, the authors can study behavioral and physiological responses to post-traumatic stress, such as avoidance and anxiety behaviors, as well as neurobiological changes and neuroplasticity, which are essential for understanding the process of PTSD and for developing potential treatments. The biological mechanisms involved in the stress response and the development of PTSD are conserved across species. As in humans, rats exhibit changes in their brain structures (such as the amygdala and hippocampus) and neurotransmitters (e.g., cortisol) following exposure to trauma. These similarities suggest that the results obtained with this model may also be relevant to understanding PTSD in humans. The predator-based stress model also provides an opportunity to study the effects of extreme stress, which may be more difficult to replicate in other models, but which is directly relevant to understanding severe trauma in humans, such as that resulting from assault, war, or other extreme traumatic events [29,30].

In the present study, we investigated the changes in behavior, levels of markers of oxidative stress, and the endocrine system (cortisol levels), following the exposure of male Wistar rats to cat urine and psychosocial stress. The use of rodents for such a study, and in this case rats, was preferred because rats have well-defined and differentiated behavior in terms of submission or attack. This is helpful in animal models of stress that are based on the induction of PTSD or depression. It is also very important to choose this species in order to obtain an animal model of PTSD, as mice require harsher conditions to develop anxious behavior [31,32]. Thus, several models are described in the literature, of which the most used are learned helplessness, time-dependent sensitization, foot shocks, underwater trauma, predator-based stress, social defeat, social isolation and chronic stress models (repeated unpredictable shock, food and water deprivation, cold swim, heat stress, shaker stress, reversal of day–night cycle, switch of cage mates) [33]. In the present study, the predator stress model was employed to facilitate the assessment of oxidative stress markers. Thus, patients with PTSD have been shown to exhibit elevated oxidative stress and increased levels of pro-inflammatory cytokines. In this context, we wanted to evaluate the effect of CBD in a predator stress model of PTSD. In studies with anxious volunteers, the repeated administration of CBD improved this condition [34]. In animal studies, the data are contradictory, with some suggesting an anti-depressant and anxiety-reducing effect [35,36], while other studies show an anxiogenic effect [37,38,39]. Another rationale for the use of CBD is its partial agonist activity at 5-HT1A receptors, a serotonin subtype implicated in the pathophysiology of PTSD. It is important to note that anhedonic behavior was not assessed in this study, and the animals’ weight did not change significantly during the study.

In the behavioral assessment, rats in the Stress + Control group exhibited increased defensive behavior in the OF test, as indicated by a longer freezing time compared to the other groups, including the Stress + CBD group, although the difference did not reach statistical significance. However, the distance traveled on the border was significantly shorter in the Stress + Control group compared to the other groups. Additionally, the rearing time—an indicator of vertical exploratory behavior—was reduced in the Stress + Control group. One study compared the effects of CBD and sertraline on PTSD-specific behavior and fear memory in mice. CBD (10 mg/kg, i.p.) improved PTSD symptoms, reduced trauma-related fear memory and anxious behaviors, and increased social interaction. These effects were observed regardless of the time of administration (pre, during or post-exposure to the aversive context). In contrast, sertraline was only effective when administered prior to the behavioral test. CBD also affected the consolidation, retrieval, and reconsolidation of fear memory, whereas sertraline only influenced retrieval [40]. One study compared the effects of CBD and CDPPB (an mGlu5 receptor agonist) on fear extinction and anxiety-like behavior in a predator odor stress model in rats. CBD did not affect fear extinction, but reduced anxiety-like behavior in the light–dark test. In contrast, CDPPB reduced conditioned fear without inducing generalized anxiety [41].

In the NOR test, regardless of the treatment or exposure to stress factors, the discrimination index was negative across all groups. However, it can be noted that the median for non-CBD treated groups is lower than in the CBD treated groups, suggesting that CBD may maintain or enhance recognition memory. The EPM test revealed no statistically significant differences between the groups, except for the time spent in the center zone. Consistent with our findings, previous studies have also reported that CBD does not significantly influence anxiety-related behaviors in the Elevated Plus Maze (EPM) test, suggesting that its anxiolytic effects may be context-dependent. Some studies reported increased ethanol consumption in response to different stress inducers, including to predator odor. However, the anxious behavior in the EPM was not associated with alcohol consumption [42,43].

To assess cognitive functions, we used the MWM test, in which the Stress + CBD group demonstrated a better performance. Thus, the time spent in the target quadrant and time spent in the location of the former platform were significantly longer. In other models of PTSD, the performance on MWM was inconsistent with some studies reporting a longer escaping time and impaired memory. A study conducted by Liu et al. showed that chronic exposure enhanced spatial learning and memory in MWM [44]. However, psychosocially stressed animals, as in this study, showed impaired memory in performing different tasks. Similar studies reported that chronic stress exposure induces impairments in recognition memory [45,46]. Studies investigating the effects of CBD on cognitive functions, particularly using the MWM test, suggest variable effects depending on the dose and duration of administration. A study conducted on mice with cerebral ischemia showed that CBD administration significantly improved performance in the MWM, suggesting that CBD could have a neuroprotective effect, supporting spatial learning and reducing neuronal degeneration in regions involved in memory, such as the hippocampus. These results are consistent with literature suggesting the potential of CBD to protect neurons against damage and improve cognitive functions under conditions of stress or brain injury [47].

In contrast, another study that administered CBD daily for 6 weeks to C57BL/6J mice did not observe significant changes in spatial learning or long-term memory in MWM. These results suggest that the effects of CBD on cognitive functions may depend on factors such as the duration of administration and the specificity of the animal model used. Thus, depending on the experimental context, CBD could have neutral or even beneficial effects on cognitive behavior [48].

Acute stress can alter memory, especially hippocampal-dependent spatial memory, through inflammatory and oxidative mechanisms. In our study, CBD-treated rats showed better performance compared to stressed controls in both tests, indicating a neuroprotective effect. This result is consistent with the observations of Campos et al. (2018) [49], who demonstrated that CBD can prevent memory impairment under acute stress conditions, and with those of Martín-Moreno et al. (2011) [50], who highlighted the beneficial effects of CBD on cognitive function in animal models of neurodegeneration. This study investigates how CBD administration prior to fear conditioning influences learning and memory in mice. The results show that CBD increased freezing during conditioning, enhanced generalized fear, and inhibited cue-dependent memory extinction. CBD also influenced dendritic plasticity in specific brain regions, suggesting an impact on learning and memory processes [51]. Another study evaluated the long-term effect of CBD on social recognition in transgenic Alzheimer’s disease model mice. CBD treatment prevented social recognition deficits without affecting anxiety or associative learning, indicating the beneficial potential of CBD in maintaining social cognitive functions [52].

CBD has been studied in several contexts for its beneficial effects on oxidative stress and immune function. One study demonstrated that CBD effectively prevents oxidative stress and stabilizes hypoxia-inducible factor-1 alpha (HIF-1α) in an animal model of global hypoxia, suggesting a protective potential under conditions of hypoxic stress [53]. Another study also evaluated the effects of CBD on vacuous chewing movements, plasma glucose levels and oxidative stress markers in rats given high-dose risperidone. The results showed that CBD significantly reduced risperidone-induced side effects, including vacuous chewing movements and changes in oxidative stress [54]. Additionally, CBD was also used in an aging study, demonstrating the ability to improve redox status and immunity in aged rats, highlighting its potential as a therapeutic strategy in combating the effects of aging. These studies suggest that CBD may play an important role in protecting cells from oxidative stress and stabilizing cellular response factors under physiological stress conditions [55,56,57].

This study also investigated the influence of CBD on oxidative stress; thus, we assayed the levels of some markers of oxidative stress such as MDA and the GSH/GSSG ratio. CBD showed a potential antioxidant effect on both matrices used for assaying MDA, while for the GSH/GSSG ratio, no statistical differences were found. This suggests that the regulation of the GSH level is more complex. Thus, exposure to stresses activates mechanisms involved in GSH metabolism, increasing the levels of the reduced form to maintain the oxidative stress at the basal level [54,58,59,60,61]. These antioxidant effects of CBD are in line with results from other studies evaluating this property [62,63].

Repeated exposures to stresses induce repeated releases of cortisol, and these spikes of cortisol may have neurotoxic effects and change behavior, memory and cognitive functions. However, in our experiment, it was observed that after 30 days, cortisol levels decreased in the Stress + Control group, as a result of increased hypothalamic–pituitary axis activity, as shown in other studies [64,65,66]. In a broad sense, we know that cortisol is a stress hormone and it is expected that when such situations occur, its secretion increases. In reality, the regulation of cortisol secretion in depression, anxiety, PTSD, etc., is more complicated. In this study, an increase in cortisol levels was observed in the Stress + CBD group, which can be interpreted as a better adaptation to stress. At moderate doses and in adaptive contexts, cortisol can facilitate cognitive coping, memory consolidation and the inhibition of disproportionate emotional reactions. Chronic exposure to situations that increase cortisol secretion is associated with cognitive deficits, affective disorders and anxiety. In this sense, it is possible that the increased cortisol levels observed after CBD administration reflect a maintenance of the functionality of cortisol secretion, temporarily depressed in states of excessive stress. By interacting with other systems and modulating other receptors (5-HT_1A_), CBD can lead to a fine-tuning of neuroendocrine responses. Thus, the increase in cortisol could correspond to the maintenance of the normal physiological response. Some studies suggest that in PTSD, cortisol levels are elevated during and immediately after exposure to stress [64], and in times when there is no stressor, cortisol levels are low. This explains the low values observed in this study in the Stress + Control group, while in the Stress + CBD group, cortisol levels increased, indicating a possible anxiolytic effect of CBD.

Taken together, these results indicated that CBD may have a positive influence on both behavioral and antioxidant metabolism, after exposure to different stresses. In adult rodents, repeated exposure to stresses induces changes in hippocampal morphology and impairments such as spatial learning and memory [67,68]. Other studies investigated the effects of psychosocial stress on neurotransmitter levels and markers of inflammation. In a study conducted by Wilson et al., the authors assayed low levels of serotonin and high levels of norepinephrine and dopamine [69]. Also, it was demonstrated that psychosocial stress is able to increase oxidative stress markers, which were reversed by other substances [70,71,72]. Although only male rodents were used in this study, it is important to acknowledge that sex may influence both the efficacy of CBD and the behavioral response to predator stress.

The findings of this study have the potential to be generalized to other species and experimental conditions to a certain extent, particularly due to the use of standardized behavioral tests and widely accepted biochemical markers of stress and oxidative balance. The behavioral assessments used in this study, namely OF, NOR, EPM, and MWM, are well-established tools in neuroscience research and have been validated across multiple species. These tests evaluate anxiety-like behavior, locomotor activity, recognition memory, and spatial learning, which are conserved across mammalian species, thus allowing potential extrapolation to other animal models. Moreover, the biochemical parameters assessed, including plasma and brain MDA levels, the GSH/GSSG ratio, and plasma cortisol concentrations, are key indicators of oxidative stress and the physiological stress response. These markers are relevant in both animal and human studies, as they reflect conserved biological pathways. The use of the predator-based PTSD model in rats effectively mimics key features of human PTSD, including anxiety and cognitive impairments.

Nonetheless, while the results offer translational value, direct generalization to humans requires caution due to interspecies differences in cognitive complexity, emotional processing, and the environmental context.

This study is not free of limitations, so some of them will be discussed below. Gender differences play an important role in stress response and CBD effects. Sex hormones, such as estrogen, can modulate the stress response and CBD metabolism, so including female rats could provide a more complete perspective on the effects of this compound in PTSD. The use of predator scent is a valid model of traumatic stress, but it does not capture the full complexity of PTSD, which can be caused by a wide range of factors (psychological trauma, chronic stress, etc.). Comparison with other models, such as social stress or repeated trauma, could improve the relevance of the results. Although the period is sufficient to observe acute and subchronic effects, PTSD is a long-term condition. Investigating the effects of CBD over a longer period or assessing whether the effects are maintained after discontinuation could add valuable information about the sustainability of the benefits. Biochemical oxidative stress assays provide information about cellular stress, but do not directly reveal the impact of CBD on neurotransmitters or brain structure. An analysis of serotonin, dopamine, GABA levels, or histological studies of the hippocampus and amygdala could complement the data obtained. The study used a single dose of CBD, and there is a possibility that responses may vary depending on the dosage. Testing different doses might help understand the dose–effect relationship. These limitations do not invalidate the study’s findings, but provide directions for improvement for future research, contributing to a deeper understanding of how CBD can be used in the treatment of PTSD. Different PTSD-inducing models with advantages and disadvantages can be seen in Table 1.

## 4. Materials and Methods

### 4.1. Animals

**Figure 7:**
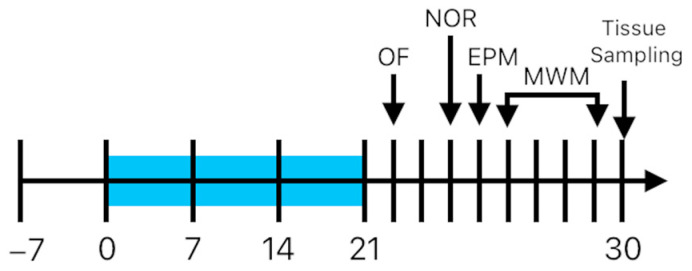
Experimental timeline. Rats were exposed to psychosocial stress between day 0 and 21. On day 10 and 20, the rats were exposed to a predator odor. CBD and vehicle were administered for 30 days. OF, open field—day 22; NOR, novel object recognition—day 24; EPM, elevated plus maze—day 25; MWM, Morris water maze—days 26–29; tissue sampling—day 30.

For this experiment, a total of 40 male 5-month-old Wistar rats (320–400 g) were used. Animals were provided by the George Emil Palade University of Medicine, Pharmacy, Science and Technology of Târgu Mureș. The randomization process was performed with the aid of a computer-based random order generator. No exclusion criteria were applied, and no animals were excluded during the experiment. Before the start of the experiment, the animals had a 7-day period of acclimation and daily handled. Animals were housed in groups of two per cage (1500 U Eurostandard Type IV S cages; 480 × 375 × 210 mm; Tecniplast SpA., Buguggiate, Italy). The animals were randomized in four groups; the Control group received the vehicle included in food in a volume of 1 mL/kg, *n* = 8, without stress exposure; the Stress + Control group received the vehicle included in food in a volume of 1 mL/kg, *n* = 8, with exposure to stress; the Stress + CBD group (CBD 10 mg/kg) received the CBD included in food in a volume of 1 mL/kg, *n* = 12, with exposure to stress; and the CBD group (CBD 10 mg/kg) received the CBD included in food in a volume of 1 mL/kg, *n* = 12, without stress exposure. A schematic timeline of the experiment can be seen in Figure 7. The laboratory conditions were standard, with a 12 h dark–light cycle, 20 ± 2 °C, 40–60% humidity, and unlimited access to food and water. For dose adjustment, the weight of the animals was measured once a week. All experimental procedures complied with European Directive 2010/63/EU and were approved by the Ethics Committee for Scientific Research of the George Emil Palade University of Medicine, Pharmacy, Science and Technology of Târgu Mureș (1851/15.09.2022).

For PTSD induction, on day 10, the rats were first exposed to a predator odor, followed by a second exposure after 10 days, on day 20. A second stressor was applied, consisting of a psychosocial stress in which the partner of each animal is changed daily.

### 4.2. Drugs and Reagents

Crystalline cannabidiol (99.5% purity from Trigal Pharma GmbH, Wien, Austria) was dissolved in sunflower oil, included in the standard pelleted rodent food, and administered daily at 12:00. Doses were calculated based on the weekly measured body weight. Vacutainer plasma tubes (BD #368856 Becton Dickinson, Basel, Switzerland) were used for collecting plasma samples. For chromatographic methods, different reagents were used, as followed: acetonitrile (VWR International, Rosny-sous-Bois, France), phosphoric acid 85% (Merck KGaA, Darmstadt, Germany), thiobarbituric acid 98%, phosphate buffer solution, trimethylpropane 99%, reduced and oxidized glutathione (GSH, GSSG), trichloroacetic acid (Sigma Aldrich, Darmstadt, Germany), sulfuric acid 98% (Chemical Company, Iași, Romania) and ultrapure water (Merck Millipore Corporation, Burlington, MA, USA). The analytical methods used for both determinations of the malondialdehyde (MDA) and GSH/GSSG ratio were previously published [72,73].

### 4.3. Behavioral Tests

The behavioral tests applied were chosen to evaluate the anxiety-like behavior, learning and memory. For the behavioral tests, no blinding method was employed.

#### 4.3.1. Open Field Test (OF)

With the aid of OF, test researchers are able to assess the general locomotor ability and anxiety-like behavior of rodents, based on the preferred position in the field. Rats were individually placed in the center of a box (60 × 60 × 50 cm) for 5 min, in order to explore the arena. The activity was recorded with the aid of a camera at 30 fps, and then analyzed with EthoVision XT (version 11.5, Noldus IT, Wageningen, The Netherlands).

#### 4.3.2. Novel Object Recognition (NOR)

**Figure 8:**
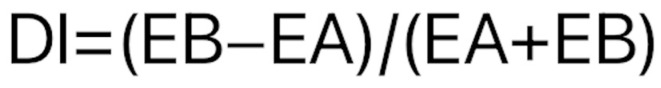
The discrimination index (DI) is the ratio between the difference between the time spent with the novel object (EB) and time spent with the familiar object (EA), and the total time spent with the objects.

This test has, at its basis, the natural behavior of the rats to explore novel objects. The test was employed on the same arena as previously described, only this time in the presence of two identical objects placed in the same location for 5 min. After that, the animals were returned to their cages. Four hours later, the test was employed again; this time, one familiar object was replaced with a novel, unknown object. Rats were placed in the same arena and let to explore the object for 5 min. The objects were glued on the arena floor. The objects were similar in size and shape but different enough in order to be distinguished. After each rat, the arena and objects were thoroughly cleaned with 70% alcohol in order to minimize the bias. The discrimination index was calculated using the formula shown in Figure 8.

#### 4.3.3. Elevated Plus Maze Test (EPM)

The assessment of the exploratory behavior was performed with the aid of the EPM test. This device comprises a plus-shaped maze, with two opposite open arms (50 × 10 cm) and two closed arms (50 × 10 × 40 cm). The distance from the floor was set at 60 cm height. The rats were placed at the crossroad, facing the open arm. After each rat, the maze was cleaned with 70% alcohol and the activity was recorded for 5 min. The time spent in open and/or closed arms and in the center zone, rearing, and head dipping were recorded [1].

#### 4.3.4. Morris Water Maze (MWM)

To assess the rats’ spatial learning and memory, the MWM test was carried out. This test comprises a circular pool, 90 cm in diameter and 60 cm deep, filled with water. A hidden platform of 9 cm in diameter was placed in a random quadrant and submerged, 1 cm below the water level. The pool was divided randomly into 4 quadrants. Visual cues were placed in the room where the test was carried out.

For 4 consecutive days, the rats underwent 4 trials a day to learn the position of the hidden platform. Each trial lasted 120 s with a 60 s intertrial period, and for each trial the position of the starting point was different in a random order. If a rat failed to find the platform, it was guided to the platform and left on the platform for 1 min. On the 5th day, the platform was removed, and the rats were placed inside the pool from the opposite quadrant to the target quadrant and left swimming for 60 s. The time spent in the target quadrant and latency to the former platform location were assayed [74,75].

### 4.4. Plasma and Brain Sampling

Rats were anesthetized with 3% isoflurane and 500 μL of peripheral blood from the tail vein were collected for determination of their basal cortisol level. At the end of the experiment, blood was collected by cardiac puncture in K3 EDTA-coated tubes and centrifuged at 3500 rpm for 10 min at 4 °C. Brain samples were collected after decapitation and washed with ice-cold phosphate buffer, weighed and immersed in liquid nitrogen. Both plasma and brain samples were stored at −80 °C until analysis.

#### 4.4.1. Oxidative Stress Parameters

For both MDA and the GSH/GSSG ratio, brains were homogenized for 5 min in IKA Ultra-Turrax Drive (Königswinter, Germany). For the MDA assay, proteins were precipitated with acetonitrile and centrifuged again at 10,000× *g* for 10 min. The resulting supernatant was diluted with pure water. To the 400 μL sample, 600 μL of thiobarbituric acid and 1000 μL of sulfuric acid were added. The mixture was heated at 100 °C for 60 min (TS-100C, Thermo-Shaker, BioSan, Riga, Latvia). For the GSH/GSSG ratio, after centrifugation, 500 μL of supernatant was mixed with 500 μL of Ellman’s reagent. GSSG samples were heated at 80 °C for 60 min. GSH samples were left at room temperature for 10 min. In both series, 300 μL of trichloroacetic acid 20% was added and centrifuged at 13,000× *g* for 10 min.

#### 4.4.2. Cortisol Assay

The quantification of the cortisol level was performed using a Rat Cortisol Kit (Arbor Assays, K003-H1, Ann Arbor, MI, USA), according to the manufacturer’s instructions.

### 4.5. Statistical Analysis

Data were analyzed with GraphPad Prism 9 (GraphPad Software, San Diego, CA, USA). The Kolmogorov–Smirnov test was used for normal distribution. Data were presented as mean ± SEM and analyzed using the one-way ANOVA test followed by Tukey’s multiple comparison test and the Kruskal–Wallis test, followed by Dunn’s multiple comparison test where appropriate. *p* < 0.05 was considered statistically significant.

## 5. Conclusions

CBD exhibited a tendency to reduce anxiety, a common symptom of PTSD, although this effect was not statistically significant. However, it demonstrated protective effects on memory, as evidenced by the MWM test. Likewise, CBD also showed a reduction in MDA levels, which implies that an improvement in PTSD symptomatology may also target a reduction in oxidative stress. Since there are several models used to induce PTSD, we cannot consider that one is better than the other, because each brings an advantage, which allows us to create a picture of the mechanisms involved in the occurrence of PTSD and how the symptoms can be improved. An important direction of this topic could be the investigation of the molecular mechanisms by which CBD influences PTSD symptoms, including the analysis of the expression of genes and proteins involved in the stress response, neuroinflammation and neuroplasticity (BDNF) and inflammatory markers (IL-6, TNF-α) in order to provide additional information about the mode of action of CBD. Another direction could be to explore the long-term effects of CBD administration, given that many current studies, including the present one, focus on immediate or short-term effects. In addition, it may be relevant to investigate the interaction between CBD and other therapeutic interventions used in PTSD, such as classical antidepressants, to determine whether the effects potentiate or interfere. On the behavioral side, it would be interesting to add specific tests of social anxiety or reactivity to aversive stimuli, to assess in more detail the impact of CBD on PTSD symptoms in various contexts. These directions could contribute to a more complete understanding of the benefits and limitations of CBD in PTSD, paving the way for its broader clinical applicability.

## Figures and Tables

**Table 1:** Advantages and disadvantages of different PTSD-inducing models.

Model	Advantages	Disadvantages
Predator-based stress	Simulates natural threats, persistent effects, activates relevant neural circuits	High variability, difficult to standardize, ethical implications
Uncontrollable electric shock	Very controllable, persistent symptoms	Physical pain, less realistic for psychological PTSD
Social stress (e.g., dominance/isolation)	Relevant for social trauma-based PTSD, induces clear behavioral changes	Effects appear slower, less intense than in Predator based stress
Variable chronic stress	Simulates cumulative stress, applicable to PTSD from prolonged exposure	Requires time to induce symptoms, harder to standardize
Traumatic sound-based stress	Relevance for war-induced PTSD (e.g., exposure to explosions), non-invasive	Does not capture all aspects of PTSD, does not induce extreme fear like Predator based stress

## Data Availability

The datasets that support the findings of this study are available from the first author upon reasonable request.

## Abbreviations

5-HT_1A_	Receptor 5-HT_1A_ for serotonin
CB1	Receptor CB1 for CBD
CBD	Cannabidiol
EPM	Elevated plus maze
GABAA	GABA A receptor
GPR55	G protein coupled receptor 55
GSH/GSSG	Ratio between reduced glutathione and oxidized glutathione
HIF-1α	Hypoxia-inducible factor-1 alpha
MDA	Malondialdehyde
MWM	Morris water maze
NOR	Novel object recognition
OF	Open field
PPARγ	Peroxisome proliferator-activated receptor gamma
PTSD	Post-traumatic stress disorder
TRPV1	Transient receptor potential for vanilloid
